# Study of Edge and Screw Dislocation Density in GaN/Al_2_O_3_ Heterostructure

**DOI:** 10.3390/ma12244205

**Published:** 2019-12-14

**Authors:** Vladimir Lucian Ene, Doru Dinescu, Iulia Zai, Nikolay Djourelov, Bogdan Stefan Vasile, Andreea Bianca Serban, Victor Leca, Ecaterina Andronescu

**Affiliations:** 1 Department of Science and Engineering of Oxide Materials and Nanomaterials, Faculty of Applied Chemistry and Materials Science, University Politehnica of Bucharest, 060042 Bucharest, Romania; vladimir.l.ene@gmail.com (V.L.E.); ecaterina.andronescu@upb.ro (E.A.); 2 Extreme Light Infrastructure-Nuclear Physics (ELI-NP), ‘Horia Hulubei’ National R&D Institute for Physics and Nuclear Engineering (IFIN-HH), 30 Reactorului Street, 077125 Măgurele, Ilfov, Romania; doru.dinescu@eli-np.ro (D.D.); iulia.zai@eli-np.ro (I.Z.); nikolay.djourelov@eli-np.ro (N.D.); andreea.serban@eli-np.ro (A.B.S.); victor.leca@eli-np.ro (V.L.); 3 Doctoral School in Engineering and Applications of Lasers and Accelerators, University Politehnica of Bucharest, 060042 Bucharest, Romania; 4 Faculty of Physics, University of Bucharest, 077125 Măgurele, Ilfov, Romania

**Keywords:** gallium nitride, epitaxial thin films, defect density, edge/screw defect, slow positrons

## Abstract

This study assesses the characteristics (edge and screw dislocation density) of a commercially available GaN/AlN/Al_2_O_3_ wafer. The heterostructure was evaluated by means of high-resolution X-ray diffraction (HR-XRD), high-resolution transmission electron microscopy (HR-TEM), and Doppler-Broadening Spectroscopy (DBS). The results were mathematically modeled to extract defect densities and defect correlation lengths in the GaN film. The structure of the GaN film, AlN buffer, Al_2_O_3_ substrate and their growth relationships were determined through HR-TEM. DBS studies were used to determine the effective positron diffusion length of the GaN film. Within the epitaxial layers, defined by a [GaN P63mc (0 0 0 2) || P63mc AlN (0 0 0 2) || (0 0 0 2) $R\bar{3}c$ Al_2_O_3_] relationship, regarding the GaN film, a strong correlation between defect densities, defect correlation lengths, and positron diffusion length was assessed. The defect densities $\rho_{d}^{e}=$ 6.13 × 10^10^ cm^−2^, $\rho_{d}^{s}=$ 1.36 × 10^10^ cm^−2^, along with the defect correlation lengths *L*^e^ = 155 nm and *L*^s^ = 229 nm found in the 289 nm layer of GaN, account for the effective positron diffusion length *L*_eff_~60 nm.

## 1. Introduction

Gallium nitride, GaN, and its alloys are mostly studied because of their usage in optoelectronic and high-temperature electronic device applications [1,2]. Different applications that require either electron [3] or hole injection [4] for different devices imply the use of either n-type or p-type semiconductors. Besides injection layers in heterostructures, GaN layers can also be used as free-standing films is applications that require “defect free” semiconductors with wide band gaps.

Positrons, the antiparticles of electrons, can easily be obtained from radioactive nuclides and normally possess high energy values. They can be used to study thin films or the areas near the surface, with one condition—they need to be slowed down, in order to obtain low-energy positrons. A method generally used to achieve lower positron energies is to place a moderator close to the positron source. One of the potential uses for defect free GaN films is in the field of positron moderation and field-assisted moderation.

Since the first trials of obtaining crystalline GaN films failed because of the high density of nitrogen vacancies, a series of developments have been made, with much work being dedicated to the fabrication of large-area heteroepitaxial GaN films on a different material platform [5]. Substrates are of great importance, because they influence to a large extent the crystal orientation, surface morphology polarity, chemical composition, and elastic strains of the grown thin films [6]. Due to the lack of commercial lattice-matched substrate, single-crystal sapphire can act as a substrate for epitaxial GaN film growth, with the best crystalline quality being obtained for the (0 0 0 1) oriented *α*-Al_2_O_3_ [7]. Despite the two crystal structures being similar and the relatively low cost of the substrate, the usage of the GaN/Al_2_O_3_ still has some limitations [8]. The lattice mismatches between the wurtzite structure of GaN and the zinc-blende structure of *α*-Al_2_O_3_ (>14%, $a_{{\mathrm{Al}}_{2}O_{3}}\;$= 4.76 Å, $a_{\mathrm{GaN}}\;$= 3.18 Å), as well as the large difference in their thermal expansion coefficients (~25%), usually determine the high density of defects and residual strain in the resulting GaN films, which affects the quality of the final devices [6,7]. Since a common goal of researchers is to increase the quality of GaN films and to extend their usage in a larger variety of applications, it is mandatory to clearly understand the types of defects present in GaN materials grown in different conditions, as well as their formation mechanisms.

Positron annihilation spectroscopy (PAS) has been established as an efficient characterization method for investigating defects at atomic level, such as vacancies, interstitials, and dislocations [9,10]. The advantage of this method is its ability to determine the type of defects [11]. Positrons, due to their positive charge, are strongly repulsed from the positive ion cores and trapped inside negatively charged or neutral defects. PAS with monoenergetic slow positrons has been used to study semiconductors, since the early 1980s [12,13]. In GaN, the point defects most probable to appear are N vacancies (V_N_) in the case of *p*-type and Ga vacancies (V_Ga_) in *n*-type GaN. Positively charged V_N_ do not trap positrons, but V_Ga_ are usually negatively charged and can therefore be observed using PAS [13,14]. On the other hand, line defects (dislocations) are less studied, even though they are of great importance for crystal growth and for the mechanical properties of the final device. There are two basic types of possible dislocations: edge and screw dislocations, which are characterized by the Burger vector being perpendicular and parallel, respectively, with respect to the dislocation line [15,16].

The main objective of the present work is to determine the density of edge and screw dislocations that determine the crystal quality of a commercially available GaN epitaxial thin film grown on Al_2_O_3_ substrate. Several aspects regarding the heterostructure, such as layer thickness, interfaces, diffusion and defects will be assessed. The positron diffusion length, derived from the slow positron experiment will be correlated with the structural features of the GaN film in order to establish if the analyzed material is suitable for positron moderation.

## 2. Materials and Methods

### 2.1. Materials

This study relies on a gallium nitride thin film grown on sapphire and purchased from NTT Advanced Technology Corporation (Kanagawa, Japan). The wafer has a high breakdown voltage, good surface uniformity and high electron mobility (>2000 cm^2^ V^−1^ s^−1^), and was grown using an epitaxial growth technique. The used wafer is further labeled as GaN/Al_2_O_3_ and has a claimed thickness of the GaN film of 300 nm. No other crucial information regarding the structure, interfaces, and defects are offered by the producer.

### 2.2. Microstructural Characterization

Advanced sample preparation has a crucial role in a successful Transmission Electron Microscopy (TEM) characterization process; therefore, the sample was first mechanically polished, then etched with an ion beam until perforation. The etching parameters were first set to a voltage of 3 kV and a current of 5 mA. After cavity formation had occurred, the ion-beam etching process continued at lower voltage and current values, with the intention of removing the produced debris and obtaining a clean area.

The GaN/Al_2_O_3_ wafer was first analyzed in order to determine its microstructure, by using a Titan Themis 200 image corrected Transmission Electron Microscope with a high-brightness XFEG electron source, produced by FEI (Hillsboro, OR, USA), coupled with a Super-X Energy Dispersive Spectroscopy (EDS) detector and a Scanning TEM (STEM) detector. The sample was investigated by High-Resolution TEM (HR-TEM) at 200 kV, Selected Area Electron Diffraction (SAED) and EDS-STEM for elemental line profiling.

Several processing and simulation software packages were used, in order to better understand the thin film’s crystal structure and lattice defects. Hence, ImageJ software (1.50b, National Institutes of Health and the Laboratory for Optical and Computational Instrumentation, Madison, WI, USA) [17] was used for processing elemental line profiles from the EDS data, while crystal structures visualization and analysis from SAED data were made with SingleCrystal^®^. The images of simulated crystals were generated from CrystalMaker^®^, a software by CrystalMaker Software Ltd., Oxford, England [18].

### 2.3. Defect Structure Determination

The GaN/Al_2_O_3_ wafer was subsequently subjected to High-Resolution X-Ray Diffraction (HR-XRD) analysis, using a 9 kW Rigaku SmartLab diffractometer (Neu-Isenburg, Germany), with a rotating Cu anode (*K*_α_ = 1.5418 Å) and a HyPix-3000 high-resolution detector, in 0D mode. The *ω*-rocking curves of selected symmetrical and asymmetrical reflections were recorded in double-axis configuration, in parallel beam mode, using a parabolic mirror (cross beam optics module) and a four bounce Ge 220 monochromator, resulting in an axial divergence of the beam of 0.003° in the vertical diffraction plane of the goniometer. To avoid the effect of sample curvature on the measurements, a narrow incidence slit (IS) of IS = 1 mm was used. Moreover, receiving slits (RS) of RS1 = 4 mm and RS2 = 38.5 mm were used on the detector side (open detector configuration), so that all diffuse scattering from the sample is taken into consideration. Measurement errors are frequent in this type of materials, mainly due to sample misalignment. Hence, the GaN/Al_2_O_3_ wafer was first aligned on the Al_2_O_3_ substrate, followed by the rocking curve measurement acquisition of the selected GaN planes.

The acquisition was recorded in the (−4°, 4°) interval, with a 0.001° step size and a speed of 1° min^−1^. Further data processing was performed using a theoretical model developed by Kaganer et al. [19], using equation:

(1) $$I\left(\omega \right)=\frac{I_{i}}{\pi }\int_{0}^{\infty }\exp\left(-Ax^{2}\ln\left(\frac{B+x}{x}\right)\right)\cos\left(\omega x\right)dx+I_{\mathrm{backgr}}$$

where *I*_i_ stands for the integrated peak intensity and *I*_backgr_ for the background intensity.

To obtain the *A* and *B* parameters, associated with the dislocation density, $\rho_{d}$, and the dislocation correlation length, *L*, respectively, a nonlinear least squares fit was performed. The values were extracted based on the minimum difference between the observed intensity and the calculated one. *A* and *B* can be defined as in the following equation:

(2) $$A=f\rho_{d}b^{2}\;;\;B=gLb^{-1},$$

Furthermore, they depend on the Burgers vector, *b*. The two non-dimensional parameters associated with the diffraction setup geometry, *f* and *g*, are determined from:

(3) $$f^{e}=\frac{0.7\cos^{2}\psi {\;\cos}^{2}\phi }{4\pi \cos^{2}\theta_{B}};\;f^{s}=\frac{0.5\sin^{2}\psi {\;\cos}^{2}\phi }{4\pi \cos^{2}\theta_{B}};\;g=\frac{2\pi \cos\theta_{B}}{\cos\phi \;\cos\psi },$$

where the angle between the scattering vector and the sample surface is ψ, the angle between the sample surface and incident vector is ϕ and the Bragg angle is $\theta_{B}$, according to the skew geometry [19].

The *f* and *g* parameters can be used to determine the density of dislocations and the characteristic correlation length for both edge (by studying an asymmetrical lattice plane) and screw (by studying a symmetrical plane) dislocations from the GaN network. When used for parameter labeling, the superscripts “e” and “s” stand for “edge” and “screw”.

### 2.4. Doppler Broadening Spectroscopy

Doppler Broadening Spectroscopy (DBS) analysis was performed at the slow positron beam line of the Institute of High Energy Physics in Beijing, China, using a HPGe detector, with a resolution of FWHM = 0.97 keV estimated for the 511 keV line. The detector was placed perpendicularly to the positron beam axis, at a distance of 20 cm from the sample. The incident positron energy was varied from *E*_+_ = 0.5 to 25 keV and the spectra were collected over a period of 8 min for a fixed *E*_+_, resulting in statistics of ~5 × 10^5^ counts in the 511 keV region.

According to Ref. [20], the implantation profile of positrons in a material with density *ρ* in g cm^−3^ can be described by:

(4) $$P\left(z,E_{+}\right)=\frac{2z}{z_{0}}\exp\left(-{\left(\frac{z}{z_{0}}\right)}^{2}\right)$$

where *z* is the depth at which the positron is located, expressed in nm, *z*_0_ = 1.13 *z*_m_, and the mean penetration depth is expressed by:

(5) $$zm=(36/\rho )E_{+}^{1.62}\;\mathrm{nm}.$$

Along with the positron implantation, several other processes occur that have to be taken into consideration when trying to solve the positron transport problem, including diffusion, drift (in the case of electric field), and free positron trapping and annihilation. The positron transport equation:

(6) $$D^{+}\frac{d^{2}c\left(z\right)}{dz^{2}}-\;\frac{d\left(v_{d}\left(z\right)c\left(z\right)\right)}{dz}-k_{t}n_{t}\left(z\right)c\left(z\right)-\lambda_{b}c\left(z\right)+I_{0}P\left(z,E_{+}\right)=0,$$

where *D*^+^ is the positron diffusion coefficient, $v_{d}\left(z\right)=\mu ℇ\left(z\right)$ is the drift velocity of the positron with mobility *µ* and electric field strength ℇ(z), *n*_t_(*z*) is the defect density, *k*_t_ is the rate constant of positron being trapped at defects, *λ*_b_ is the bulk annihilation rate, *I*_0_ is the intensity of the implanted positrons, also known as the thermal positrons equilibrium, can be used to determine the time averaged positron density *c*(*z*) at a certain depth [21].

Differences in density of the layers in a structure are taken into account by the use of a modified positron implantation profile described by:

(7) $$P_{\rho }\left(z_{\rho },E_{+}\right)=\rho \left(z_{\rho }\right)/\rho_{0}P\left(z,E_{+}\right),$$

where $z=\int_{0}^{z_{\rho }}\rho \left(\zeta \right)/\rho_{0}d\zeta$ and *ρ*_0_ is the density of the substrate. In the analysis of the experimental data, the values of 6.15, 3.98, and 3.26 g cm^−3^ were used for the densities of GaN, AlN, and Al_2_O_3_, correspondingly.

The effective positron diffusion length, *L*_eff_, is limited by the defect density and is described by the equation:

(8) $$L_{\mathrm{eff}}=\left[D^{+}/\left(k_{t}n_{t}+\lambda_{b}\right)\right],$$

where the product between the defect density and the positron trapping rate, *k*_t_*n*_t_, for vacancies, usually holds the value of 10^15^ s^−1^.

The shape of the 511 keV annihilation line was analyzed by evaluating the sharpness (*S*) parameter. The *S* parameter is defined as the sum of counts in the central region (|*E*_γ_ − 511| < 0.78 keV) of the peak, relative to the total peak counts, and accounts for the low-momentum of valence electrons within the material. Because of the dependency between the mean e^+^ implantation depth, *z*_m_, and *E*_+_, the experimental data *S*(*E*_+_) represents a depth profile.

The *S*(*E*_+_) can be fitted using a model described by:

(9) $$S\left(E_{+}\right)=S_{e}F_{e}(E_{+})+S_{s}F_{s}(E_{+})+\sum S_{i}F_{i}(E_{+}),$$

with $F_{e}(E_{+})+F_{s}(E_{+})+\sum F_{i}(E_{+})=1$, where *F*_e_(*E*_+_) is the fraction of epithermal (non-thermalized) positrons annihilated at the surface, and *F*_s_(*E*_+_) and *F*_i_(*E*_+_) are the fractions of thermalized positrons annihilated at the surface, and in the i-th layer, respectively. *S*_e_, *S*_s_, and *S*_i_ are annihilation characteristic parameters for the epithermal positrons at the surface, thermalized positrons at the surface, and annihilated positrons in the i-th layer.

VEPFIT software uses discretization as a fast method for the numerical solution of the positron transport equation (Equation (6)). A semi-linear fitting procedure is based on the least squares method to quantify the goodness of fit for the model parameters. This solves a linear minimization problem in one step (for the set of *S* parameters) and a non-linear problem (to obtain the fractions of the annihilated positrons, *F*_e_(*E*_+_), *F*_s_(*E*_+_), and *F*_i_(*E*_+_), that depend on non-linear parameters like layer boundaries and diffusion lengths) iteratively in several steps. The linear problem is solved within every non-linear iteration step. Detailed description of the fitting can be found in [20].

## 3. Results and Discussion

### 3.1. Microstructural Characterization

After analyzing the microstructure of the sample in cross-section, the existence of an aluminum nitride, AlN, buffer layer was observed, with a measured thickness of 21 nm. Literature studies also report this approach of accommodating the GaN network to that of the substrate, in this case Al_2_O_3_. By using such a buffer, the strain and the number of defects in the GaN film generated during growth processes, are reduced [22].

**Figure 1:**
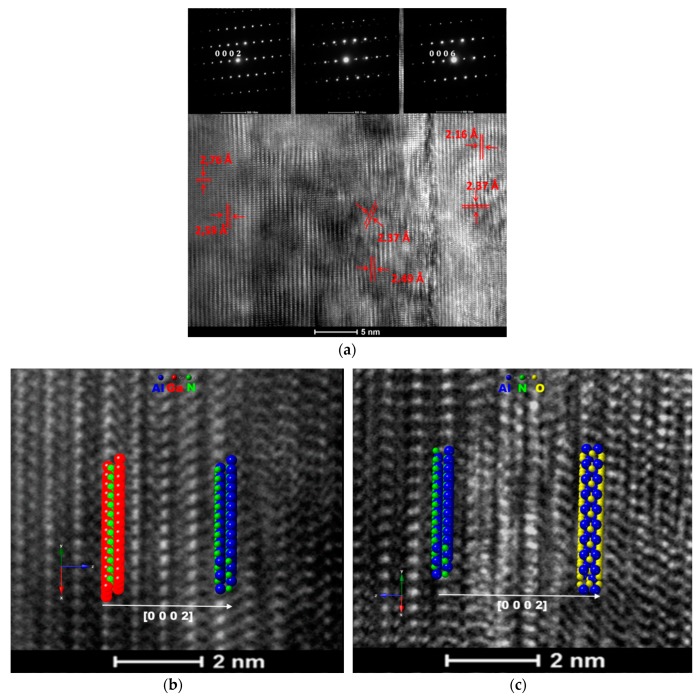
HR-TEM micrographs showing the display of atom planes in respect to their corresponding interfaces for: (**a**) GaN/AlN/Al_2_O_3_, (**b**) GaN/AlN—interface, (**c**) AlN/Al_2_O_3_ interface. The insets show the corresponding SAED patterns.

Figure 1 shows the TEM micrographs and SAED patterns (see the insets) of planes near the AlN/GaN interface and near the Al_2_O_3_/AlN interface in the heterostructure (Figure 1a), respectively, with overlays of simulated crystals (Figure 1b,c).

In the right side of Figure 1a, the interplanary distances were measured as 2.16 Å and 2.37 Å, corresponding to (0 0 0 6) and ($1\;1\;\bar{2}\;0$) planes, confirming the $R\bar{3}c$ rhombohedral structure of Al_2_O_3_ (ICDD 00-046-1212). The middle of the figure shows interplanary distances of 2.49 Å and 2.37 Å, corresponding to (0 0 0 2) and ($2\;0\;\bar{2}\;2$) planes, confirming the *P63mc* hexagonal structure of AlN (ICDD 00-025-1133). The SAED patterns attributed to each analyzed area suggest the existence of a semi-coherent interface, corresponding to hexagonal AlN grown over the rhombohedral Al_2_O_3_ substrate, with the relationship $R\bar{3}c$ Al_2_O_3_ (0 0 0 2) || (0 0 0 2) AlN *P63mc,* as seen in Figure 1c. This area is about 1 nm wide and contains point defects and a high density of dislocations. On the left side of Figure 1a, interplanary distances of 2.59 Å and 2.76 Å, corresponding to (0 0 0 2) and ($1\;0\;\bar{1}\;0$) planes, confirm the *P63mc* hexagonal structure of *P63mc* GaN (ICDD 00-050-0792). It can be deduced that through a coherent interface, hexagonal GaN has grown over the hexagonal AlN with a growth relationship that can be expressed as *P63mc* GaN (0 0 0 2) || (0 0 0 2) AlN *P63mc*, as seen in Figure 1b.

It appears that at the proximity of the interface between the substrate and the buffer, the Al_2_O_3_ manifests a disordered microstructure and because of the substrate effect on the buffer, a high density of dislocations can be found inside the AlN structure. A slight nitrogen diffusion outside the AlN buffer boundary towards the substrate can be identified, suggesting an exchange of oxygen with nitrogen atoms at the surface of the sapphire (nitridation of the sapphire substrate), which might locally affect the crystallographic relationship of growth, a phenomenon similar with the findings of Claudel et al. [23] and Miyagawa et al. [24]. Their theory was based on the formation of phase inversion domains (ID) that usually occurs when an Al(O,N) interlayer is present. Since no such interlayer was identified by TEM means in our case, the potential presence of ID might be associated with screw dislocations formed during the growth process. It is worth mentioning that no voids, defects, or separation between AlN and GaN were detected along their interface, due to the fact that their networks have the same structure with similar lattice parameters, meaning that the nitride layers were continuous and well-adhered to each other, although defects from the substrate may carry on through the film, as recently indicated also by Jimenez group [25].

**Figure 2:**
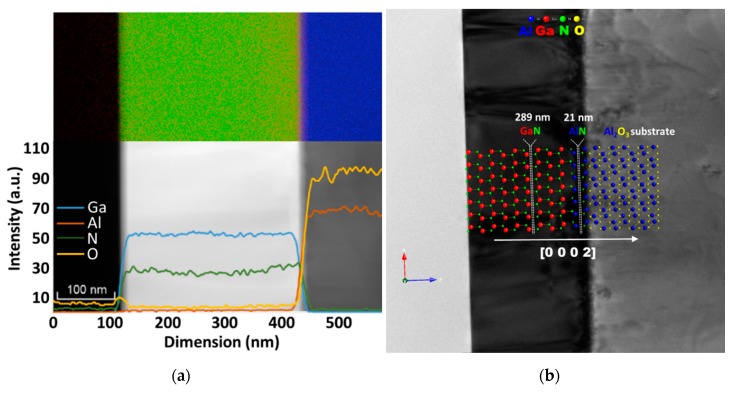
STEM with EDS and line profile image of the GaN/AlN/Al_2_O_3_ (**a**) and TEM micrograph with simulated crystals showing the overview of the wafer (**b**).

The thicknesses of the layers were measured by STEM (Figure 2a) with values of 289 nm and 21 nm for the GaN film and the AlN buffer, respectively. The small thickness of the buffer layer was to be expected, given that the Al adatoms on the C-face have higher mobility and thus shorter time available for incorporation into the lattice, as already presented by Li et al. [26], who studied the AlN growth on different face sapphire substrates. The overall structure of the heterostructure is illustrated in Figure 2b, showing the characteristic growth relationship *P63mc* GaN (0 0 0 2) || (0 0 0 2) AlN *P63mc* || (0 0 0 2) Al_2_O_3_ $R\bar{3}c$.

**Figure 3:**
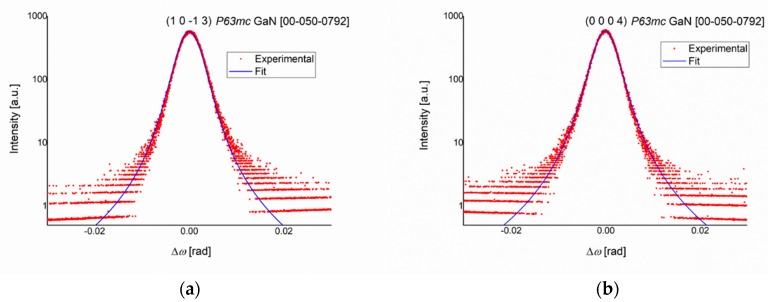
Rocking curves (*ω* scans) around (**a**) the ($1\;0\;\bar{1}\;3$) plane and (**b**) the (0 0 0 4) plane of the GaN film.

In the process of evaluating the defect correlation length and the dislocation density of the GaN network, two rocking curves (*ω* scans) were measured, and the results are presented in Figure 3. To determine the edge characteristics $\rho_{d}^{e}$ and *L*^e^, the ($1\;0\;\bar{1}\;3$) plane of GaN was taken into account, while for the screw values $\rho_{d}^{s}$ and *L*^s^ assessment, the (0 0 0 4) plane was used.

The mathematical interpretation of the data implies a simple intensity distribution that is described by a one-dimensional integral (Equation (1)). This fit is made possible by using an open detector, which accounts for the high intensity of the diffraction pattern. This model cannot be applied on overlapping peaks. For the latter case, a fit of a two-dimensional integral is possible, when the diffraction configuration is adjusted with an analyzer at the detector side (triple-axis) for a better separation of the peaks. To reduce the complexity of the calculations, the ($1\;0\;\bar{1}\;3$) and (0 0 0 4) planes were chosen because of the lack of overlapping diffraction interferences near their respective *ω* (32.102° and 36.401°, respectively).

The length of the Burgers vector of edge dislocations was *b*^e^ = 0.32 nm, and for the screw dislocations, *b*^s^ = 0.52 nm. Parameters *f*^e^ and *g*^e^, *f*^s^ and *g*^s^, along with a series of defect densities and defect correlation lengths, were calculated, and the results are summarized in Table 1.

By analyzing the obtained results, it was confirmed that edge and screw dislocations were present in the GaN/AlN/Al_2_O_3_ wafer, and the high values for the defect correlation lengths suggest that the dislocations present at the AlN/Al_2_O_3_ interface can easily glide along, towards the GaN film, covering a distance comparable with the GaN film thickness, as the TEM analysis also pointed out. Because the applied model can only quantify pure edge and screw dislocations, this study will not take into account the contributions of mixed dislocations.

### 3.2. Positron Annihilation Data

**Figure 4:**
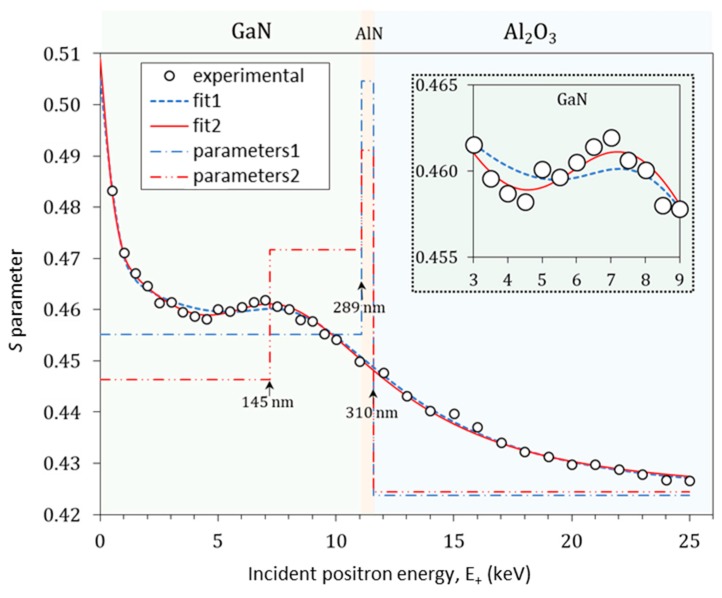
Plotted depth profiles *S*(*E*_+_) of GaN/AlN/Al_2_O_3_ heterostructure. The experimental errors are in the order of the experimental point size. The stairs labeled as parameters1 and parameters2 represent the best *S* parameters obtained by the fit of a 3-layer model and a 4-layer model, respectively, to the experimental data by the VEPFIT software. The same fit also gives the best effective positron diffusion lengths *L*_eff_, summarized in Table 2. The inset shows a zoom for the incident positron energies between 3 and 9 keV.

Figure 4 shows the depth profiles *S*(*E*_+_) obtained for the GaN/AlN/Al_2_O_3_ heterostructure. The layer boundary depths are calculated by Equation (5) and indicated in the figure. The sharp decrease in *S* for *E*_+_ ≲ 1 keV is due to the annihilated epithermal positrons. Because of their high kinetic energy in the moment of annihilation, *S*_e_ does not reflect the material structure. At *E*_+_ ≳ 1 keV, a slower decrease of *S* with *E*_+_ is observed. Since Positronium (Ps) is formed only at the GaN surface and not in the bulk [27] and the self-annihilation of its singlet form, *p*-Ps, contributes to the sharpening of the Doppler broadened annihilation line, the *S*_s_ is larger than *S*_GaN_ in the bulk of GaN film. This, along with the back-diffusion of thermalized positrons to the surface, explain the slow decrease. Between ≈4 and ≈8 keV, the greater fraction of the positrons annihilates in the GaN film, as can be seen by the observed plateau in *S*(*E*_+_). In the next region, *E*_+_ > 8 keV, a decrease in *S* follows, which tends to reach a saturation level. Full saturation can be expected at *E*_+_ > 25 keV, a high enough energy to annihilate all implanted positrons entirely in the Al_2_O_3_ substrate. The shape of the upper region of the plateau (6 keV < *E*_+_ < 8 keV) shows slightly bigger *S*, which can be attributed to the presence of the AlN buffer layer.

The thickness of the layers determined by the TEM analysis was fixed in the VEPFIT analysis. The diffusion length in the substrate was also fixed to 80 nm in accordance with the available literature data for sapphire [28]. Using VEPFIT, a first analysis (labeled as fit1 in Figure 4) was performed with a three-layer model, i.e., one for the 289-nm-thick GaN, a second for the 21-nm-thick AlN, and a third for the sapphire substrate. The characteristic *S* parameters and effective positron diffusion lengths, *L*_eff_, of the different layers obtained from the best fit are summarized in Table 2 and also plotted as stairs (labeled as parameters1) in Figure 4. It should be mentioned that perfect lattice semiconductors are expected to have *L*_eff_ in the range 200–300 nm [29]. The longest effective positron diffusion length *L*_eff_ = 92 ± 3 nm for GaN was obtained for a hydride vapor phase epitaxy (HVPE) GaN [30]. By comparing their experimental (*S*, *W*) results with the theoretically calculated one, the authors concluded that the HVPE-GaN sample is defect free. In the present case, the GaN’s effective diffusion length obtained by fit1 was found to be *L*_eff_ = 63 ± 3 nm. According to Equation (8), the higher the defect density, the shorter the positron diffusion length. Short positron diffusion length for GaN can be caused by the positron interaction with dislocations. Even though the dislocations are considered to be shallow traps, they can reduce *L*_eff_ by enhanced scattering of thermal positrons on them. The dislocations can even become deep traps, due to the tendency of vacancies to accumulate along them, enhancing the negative charge densities [13], therefore trapping positrons more easily. Based on this theory, the short effective positron diffusion length is in accordance with the high dislocation densities near the GaN/AlN interface determined by both TEM analysis and XRD defect assessment.

The normalized chi-square value for fit1 is rather high, *χ*^2^ = 2.07, which indicates that the chosen model is not good enough to explain the experimental data. As can be seen in the inset in Figure 4, the high *χ*^2^ comes from the plateau region. Two hypotheses were tested in the search for a better model.

The first hypothesis is that the GaN film is not homogenous. The analysis by VEPFIT was performed with a 4-layer model (see fit2 in Figure 4). The GaN film was split into two sublayers. The curve of fit2 matches well to the experimental points, which is also reflected in an improved goodness of fit *χ*^2^ = 1.48 (see Table 2) compared to fit1. The effective positron diffusion length in the first GaN sublayer is found to be *L*_eff_ = 60 ± 3 nm, a value close to what was obtained by fit1. The *L*_eff_ = 46 ± 9 nm for the second GaN sublayer is shorter than the first sublayer value, which indicates the lower quality of GaN, i.e., the higher defect density. When a positron is trapped at a defect, the probability of annihilating it with core electrons, which possess high momentum, is decreased compared to a defect-free sample [12]. This results in smaller broadening of the annihilation line and thus in a bigger *S* parameter. However, a bigger *S* parameter could mean not only higher defect density, but also larger defects or a higher Ps yield in Ps-forming materials. The *S* parameter value of 0.472 ± 0.003 (see Table 2) for the second GaN sublayer is bigger than 0.446 ± 0.002 found for the first GaN sublayer. Since Ps is not formed in bulk GaN [27], we can draw the conclusion that the GaN film of the GaN/AlN/Al_2_O_3_ wafer is not homogenous in depth. Most probably, the non-homogeneity is due to the high concentration of dislocations in the GaN close to the GaN/AlN interface. This observation is also in agreement with the elemental analysis provided by TEM (Figure 2a), which points out that the growth of the GaN film occurred with a slight decrease in the N content, suggesting a slight stoichiometry shift from the interface with the AlN buffer towards the surface of the film.

The above VEPFIT analysis did not take into consideration any electric field between the two semiconductor GaN and AlN layers. A charge density present at the interface sites, generates an internal electric field that influences the positron transport through the heterojunction interface. The second hypothesis is based on this phenomenon. Equalization of the Fermi levels by charge transfer between two materials is responsible for the formation of a potential barrier [29]. The potential barrier of +0.27 eV is calculated from the difference in the positron affinities in the two materials [31]. Actually, the positrons cannot diffuse equally well in both directions across the interface. The diffusing positrons in the GaN layer are pushed back by the potential barrier at the interface, while those which diffuse in AlN will fall in a potential well. The positrons tend to localize at the well barrier interface in a nitride heterostructure, as shown by theoretical calculations [32]. To compensate for these effects, which can lead to incorrect estimation of both the thickness of the layers and the effective positron diffusion lengths by VEPFIT analysis, Hu et al. introduced a strong electric field associated with a GaN/SiC interface in order to mimic the barrier effect in VEPFIT [33]. The same approach was applied in the present study for the GaN/AlN interface. By increasing the strength of the electric field up to 1 × 10^5^ V cm^−1^, the goodness of fit (these additional fits are not shown) was improved to about *χ*^2^ = 1.7; however, no significant change in the effective positron diffusion lengths in GaN was observed. Therefore, the GaN film in the studied wafer cannot be considered to be a defect-free material, implying limitations on its use as positron moderator, due to a rather high amount of dislocations found inside the film.

## 4. Conclusions

A commercially available GaN/AlN/Al_2_O_3_ epitaxial wafer was qualitatively and quantitatively characterized by means of TEM, XRD and DBS studies. Due to a high mismatch between the Al_2_O_3_ substrate and the AlN interlayer, both point defects and dislocations were found in the structure of the film. Within the epitaxial layers, defined by a [GaN P63mc (0 0 0 2) || P63mc AlN (0 0 0 2) || (0 0 0 2) $R\bar{3}c$ Al_2_O_3_] relationship, a strong correlation between dislocation densities, defect correlation lengths and effective positron diffusion length was assessed. The dislocation densities $\rho_{d}^{e}\;=$ 6.13 × 10^10^ cm^−2^, $\rho_{d}^{s}=$ 1.36 × 10^10^ cm^−2^, along with the defect correlation length *L*^e^ = 155 nm and *L*^s^ = 229 nm found in the 289 nm layer of GaN, account for the relatively short effective positron diffusion length *L*_eff_~60 nm.

## Figures and Tables

**Table 1:** Defect densities and defect correlation lengths for GaN in the GaN/AlN/Al_2_O_3_ wafer.

Plane	$\rho_{d}^{e}\;({\mathrm{cm}}^{-2})$	$\rho_{d}^{s}\;({\mathrm{cm}}^{-2})$	*L*^e^ (nm)	*L*^s^ (nm)
(0 0 0 4)	-	1.36 × 10^10^	-	229
($1\;0\;\bar{1}\;3$)	6.13 × 10^10^	-	155	-

**Table 2:** Best fit parameters obtained by VEPFIT of the *S*(*E*_+_) depth profiles. The values without error margins were considered fixed parameters in the model.

		Fit1 *χ*^2^ = 2.07			Fit2 *χ*^2^ = 1.48		
Layer/Sublayer		*L*_eff_ [nm]	*S*	*d* [nm]	*L*_eff_ [nm]	*S*	*d* [nm]
GaN	1	63 (3)	0.4552 (7)	289	60 (3)	0.446 (2)	145
	2				46 (9)	0.472 (3)	144
AlN		24 (1)	0.546 (3)	21	43 (8)	0.49 (2)	21
Al_2_O_3_		80	0.4238 (3)	-	80	0.4244 (3)	-
